# Predicting Unscheduled Emergency Department Revisits Leading to Acute Hospital Admissions Among Older Adults

**DOI:** 10.1093/geroni/igab046.2233

**Published:** 2021-12-17

**Authors:** Tony Rosen, Yufang Huang, Matthew McCarty, Michael Stern, Yiye Zhang, Daniel Barchi, Rahul Sharma, Peter Steel

**Affiliations:** 1 Weill Cornell Medical College / NewYork-Presbyterian Hospital, PELHAM, New York, United States; 2 Weill Cornell Medicine, New York, New York, United States; 3 NewYork-Presbyterian Hospital Weill Cornell Medical Center, New York, New York, United States; 4 Weill Cornell Medical College, New York, New York, United States; 5 NewYork-Presbyterian Hospital, New York, New York, United States

## Abstract

Background: Unscheduled emergency department (ED) revisits leading to acute hospital admission (RVA) are tantamount to a failed discharge, associated with physician error, mis-prognosis, and inadequate care planning. Previous research has shown RVA to be associated with adverse outcomes such as ICU admissions, long hospitalizations and mortality. Given the limited impact of pre-existing screening tools for older adults, we developed and validated a machine learning model to predict individual patient risk of RVA within 72 hours and 9 days of index ED visits. Method: A machine learning model was applied to retrospective electronic health record (EHR) data of patients presenting to 2 geographically and demographically divergent urban EDs in 2019. 478 clinically meaningful EHR data variables were included: socio-demographics, ED and comorbidity diagnoses, therapeutics, laboratory test orders and test results, diagnostic imaging test orders, vital signs, and utilization and operational data. Multiple machine learning algorithms were constructed; models were compared against a pre-existing adult ED-RVA risk score as a baseline. Results: A total of 62,154 patients were included in the analysis, with 508 (0.82%) and 889 (1.4%) having 72-hour and 9-day RVA. The best-performing model, combining deep significance clustering (DICE) and regularized logistic regression, achieved AUC of 0.86 and 0.79 for 72-hour and 9-day ED-RVA for older adult patients, respectively, outperforming the pre-existing RVA risk score (0.704 and 0.694). Discussion: Machine learning models to screen for and predict older adults at high-risk for ED-RVA may be useful in directing interventions to reduce adverse events in older adults discharged from the ED.

